# Health Care Provider Stigma Toward Patients With Substance Use Disorders: Protocol for a Nationally Representative Survey

**DOI:** 10.2196/47548

**Published:** 2023-09-26

**Authors:** Carrigan Leigh Parish, Daniel J Feaster, Harold A Pollack, Viviana E Horigian, Xiaoming Wang, Petra Jacobs, Margaret R Pereyra, Christina Drymon, Elizabeth Allen, Lauren K Gooden, Carlos del Rio, Lisa R Metsch

**Affiliations:** 1 Department of Sociomedical Sciences Mailman School of Public Health Columbia University Miami, FL United States; 2 Department of Biostatistics Miller School of Medicine University of Miami Miami, FL United States; 3 Crown Family School of Social Work, Policy, and Practice University of Chicago Chicago, IL United States; 4 Department of Public Health Sciences Miller School of Medicine University of Miami Miami, FL United States; 5 Office of Behavioral and Social Clinical Trials Division of Behavioral and Social Research National Institute on Aging/National Institute of Health Bethesda, MD United States; 6 National Opinion Research Center Chicago, IL United States; 7 Division of Infectious Diseases Department of Medicine Emory University School of Medicine Atlanta, GA United States; 8 Department of Sociomedical Sciences Mailman School of Public Health Columbia University New York, NY United States; 9 School of General Studies Columbia University New York, NY United States

**Keywords:** substance use disorders, provider stigma, cross-sectional survey study, provider attitudes, survey methodology

## Abstract

**Background:**

The US overdose epidemic is an escalating public health emergency, accounting for over 100,000 deaths annually. Despite the availability of medications for opioid use disorders, provider-level barriers, such as negative attitudes, exacerbate the treatment gap in clinical care settings. Assessing the prevalence and intensity of provider stigma, defined as the negative perceptions and behaviors that providers embody and enact toward patients with substance use disorders, across providers with different specialties, is critical to expanding the delivery of substance use treatment.

**Objective:**

To thoroughly understand provider stigma toward patients with substance use disorders, we conducted a nationwide survey of emergency medicine and primary care physicians and dentists using a questionnaire designed to reveal how widely and intensely provider attitudes and stigma can impact these providers’ clinical practices in caring for their patients. The survey also queried providers’ stigma and clinical practices toward other chronic conditions, which can then be compared with their stigma and practices related to substance use disorders.

**Methods:**

Our cross-sectional survey was mailed to a nationally representative sample of primary care physicians, emergency medicine physicians, and dentists (N=3011), obtained by American Medical Association and American Dental Association licensees based on specified selection criteria. We oversampled nonmetropolitan practice areas, given the potential differences in provider stigma and available resources in these regions compared with metropolitan areas. Data collection followed a recommended series of contacts with participants per the Dillman Total Design Method, with mixed-modality options offered (email, mail, fax, and phone). A gradually increasing compensation scale (maximum US$250) was implemented to recruit chronic nonresponders and assess the association between requiring higher incentives to participate and providers stigma. The primary outcome, provider stigma, was measured using the Medical Condition Regard Scale, which inquired about participants’ views on substance use and other chronic conditions. Additional survey measures included familiarity and social engagement with people with substance use disorders; clinical practices (screening, treating, and referring for a range of chronic conditions); subjective norms and social desirability; knowledge and prior education; and descriptions of their patient populations.

**Results:**

Data collection was facilitated through collaboration with the National Opinion Research Center between October 2020 and October 2022. The overall Council of American Survey Research Organizations completion rate was 53.62% (1240/2312.7; physicians overall: 855/1681.9, 50.83% [primary care physicians: 506/1081.3, 46.79%; emergency medicine physicians: 349/599.8, 58.2%]; dentists: 385/627.1, 61.4%). The ineligibility rate among those screened is applied to those not screened, causing denominators to include fractional numbers.

**Conclusions:**

Using systematically quantified data on the prevalence and intensity of provider stigma toward substance use disorders in health care, we can provide evidence-based improvement strategies and policies to inform the development and implementation of stigma-reduction interventions for providers to address their perceptions and treatment of substance use.

**International Registered Report Identifier (IRRID):**

DERR1-10.2196/47548

## Introduction

### Background

The US overdose epidemic is a public health emergency that continues to escalate, accounting for over 100,000 deaths annually [1]. Fueled largely by the use of prescription opioids, heroin, and synthetic opioids other than methadone (primarily fentanyl) [2-4], which contributed to 75% of overdose deaths in 2021, the epidemic has been further intensified by rises in stimulant-related overdose deaths, particularly those linked with cocaine and psychostimulants [5]. Rising cocaine and psychostimulant-related overdose deaths also often involve opioid use [6-8], and polysubstance use involving both stimulants and opioids is a growing pattern that warrants focused interventions [9]. In addition, the accidental overdose epidemic has been attributed to “multiple distinctive sub-epidemics of different drugs” that have treatments that are different, such as the availability of medications for opioid use disorder (MOUD) versus the range of behavioral health services to treat stimulant use [7,10].

National attention to this overdose epidemic highlights the need for resources for health care providers to enhance the prevention and treatment efforts toward substance use disorders [7], including provider training and integration of substance use treatment within current health care systems. Barriers may arise from providers’ negative attitudes toward persons with substance use disorders [9,10], including provider stigma, defined as the negative attitudes, perceptions, and behaviors that providers embody and enact toward their patients either subtly or involuntarily [11,12]. Provider stigma is increasingly recognized as an important and understudied barrier to the effective treatment and prevention of substance use [13-15], as well as one that is notably complex. Given that it encompasses stigma professionally and personally (eg, applying labeling, stereotypes, discrimination, and social marginalization) [16], provider stigma is one of the more substantive forms of stigma that people with substance use disorders may experience, even compared with friends, family, and coworkers, and often manifests as facing scrutiny and skepticism, treatment refusal, and delayed or substandard treatment [17-19]. There are also many causes of provider stigma, including negative perceptions and attitudes (eg, viewing the “illness ahead of the person”), compassion fatigue and professional burnout, biases (both conscious and unconscious), therapeutic pessimism and helplessness (eg, negative outlook on the likely benefits of treatment), and inadequate training and skills [20-26]. As such, research efforts that focus on quantifying and addressing the scope of provider stigma toward persons who use drugs is a critical first step in enhancing the health care response to expanding the delivery of substance use treatment [27-29].

### Key Scientific Objectives of Clinical Trials Network 0104

Providers are increasingly called upon to identify patients with substance use disorders and refer patients to MOUD and other treatment resources [16,30]. Thus, understanding provider stigma and attitudes toward patient substance use is especially important in developing interventions to expand access to substance use treatment. The specific aims of this study are as follows:

1. Define the scope of provider stigma toward substance use by substance type and compare this stigma to provider stigmas directed at other medical conditions.
2. Compare providers’ screening, treatment, and referral practices for substance use with their screening, treatment, and referral practices for other medical conditions.
3. Identify factors related to the delivery of substance use treatment in US-based primary care and emergency medicine settings to inform educational and intervention strategies addressing provider stigma.

## Methods

### Study Overview and Design

This study examines provider stigma by conducting a cross-sectional national provider survey of general practice, emergency medicine, and the dental workforces (October 2020 to October 2022). A representative sample of physicians and dentists was surveyed to better understand their attitudes and stigma toward patients with substance use disorders, and the role that these attitudes and stigma play in shaping providers’ screening, treatment, and referral practices concerning the provision of substance use treatment.

### Ethics Approval

The study was reviewed and approved by the single Institutional Review Board (IRB) at the University of Miami (UM; IRB# IRB00010711) that provided regulatory oversight over all the participating sites. Consent forms were not used because of the nature of the study, which involved minimal participant risks. Instead, we received a waiver of informed consent from the UM IRB, whereby the cover letter provided the required consent information needed to help inform the participant’s decision to participate. Consent was, therefore, not documented in writing, but rather implied through survey completion. Opt-out, passive consent methods have been shown to be more cost-efficient and feasible, without violating participants’ autonomy or essential interests [31-33]. Confidentiality was maintained using unique participant ID numbers. The master list linking participant ID numbers to participant contact information was maintained at a secure location by the designated staff for recruitment purposes. At the end of data collection, only deidentified study data were made available to the study investigators. Compensation for participation was issued, as described in further detail in the section on incentives.

### Participants

#### Overview

The study population consisted of primary care physicians, emergency medicine physicians, and dentists. Given their regular contact with patients with substance use disorders, these providers are well positioned to either refer for or deliver substance use treatment within their routine clinical care practices [34]. We now detail the rationale for including each provider group.

#### Primary Care Physicians

Efforts to expand substance use treatment have largely turned to primary care settings, especially to reach patient populations that heavily rely on public health care systems (eg, those who are homeless and have low income) and that present with multiple comorbidities (eg, those with substance use and HIV) [35-37]. Recent stigma studies among primary care physicians have largely focused on opioids, particularly their attitudes toward patient opioid use and their clinical practices related to prescribing MOUD. These studies have documented negative attitudes toward persons with opioid use disorders (OUD) as well as associations between higher provider stigma and minimal offering of OUD treatment practices and referrals for the treatment of OUD in these settings [13,38-40].

Although the practice of primary care is described as the services provided from a patient’s first point of “entry” on through their continued comprehensive care, primary care also routinely involves collaborating with other health care providers, including consultations and referrals [41]. Therefore, we sought to survey all primary care specialties as defined by the American Medical Association (AMA), including family medicine, general practice, internal medicine, obstetrics/gynecology (OB/GYN), and pediatrics [42]. With regard to the field of OB/GYN medicine, preventive gynecologic appointments (eg, Papanicolaou smears) often represent women’s only contact with a medical care provider and thus are the only opportunity for these women to be screened and treated for other medical conditions [43-45]. A 2017 study found that around half of women either identify their OB/GYN as their primary care provider or do not have a primary care provider [46]. In addition, in the field of pediatrics, substance use is increasingly prevalent among youth and young adults; opioid misuse is being initiated at a young age, increasing the risks for future substance use disorders [47-52], whereas stimulant use (eg, methamphetamine and inhalants) is also increasing at striking rates, with about half of graduating high school students reporting the use of a stimulant at least once [53,54]. In response, the American Academy of Pediatrics has advocated the expanded use of substance use treatment for youth and adolescents and called upon pediatricians to intervene early and help prevent potential substance use among their patients [55].

Finally, as specialists in internal medicine, infectious disease physicians were specifically included in our sample, as they also often provide primary care treatment for patients with HIV, especially as HIV treatment has evolved from a fatal disease to a manageable chronic condition that warrants screening for other major medical comorbidities [56-58]. A 2018 study showed that three-fourths of infectious disease physicians also act as the primary care physicians for patients with HIV [59]. Studies suggest that the increasing rates of infectious diseases such as HIV/AIDS are driven by the opioid crisis and that integrated substance use disorder and infectious disease treatment can reduce substance misuse and infectious disease transmission, respectively [60]. The National Institute of Allergy and Infectious Diseases and the Institute of Human Virology have underscored the significant role of infectious disease providers in addressing substance use among patients in their practices [61].

#### Emergency Medicine Physicians

Emergency medicine physicians were included in the study population, given that they frequently encounter patients with substance use disorders, especially those at high risk for overdose, trauma, or suicide [62-64]. These patients often do not regularly seek primary care and instead use and rely on emergency departments for medical treatment [65,66]. Prior studies assessing emergency medicine physicians’ regard for patients with substance use disorders have featured small sample sizes, and their findings were often limited in generalizability [17,62]. Overall, these studies documented low regard for patients with substance use disorders by emergency medicine physicians compared with patients with other medical conditions [17]. Studies have suggested that such negative attitudes can be mitigated by providing physicians with evidence-based trainings [62].

#### Dentists

The professional role of dentists has evolved into one that is more medically focused and encompasses limited preventive primary care, including medical chairside screenings [67]. As health care providers who regularly manage and treat patients with dental pain, dentists encounter challenges analogous to those of other health care practitioners who encounter and treat patients who live with substance use disorders [68-70]. Dentists have reported concerns that patients are using substances, yet they often do not routinely query patients about it [71-74]. Dentists are responsible to treat many oral implications of substance use, such as rampant caries, xerostomia, tooth loss, and the pathognomonic and overt dental disease known as “meth mouth” [75,76]. However, dentists across the United States have reported minimal, if any, prior training and knowledge in the area of patient substance use [73]. As such, the American Dental Association (ADA) has called for dentists to obtain continuing education in opioid-related practices and use brief interventions to encourage and refer patients for opioid use treatment [77]. However, there are limited data regarding dentists’ stigma and attitudes toward addressing substance use in their clinical practices.

### Eligibility Criteria

Study participants were required to (1) currently be in clinical practice (and planning to remain so for the next year) as a primary care or emergency medicine physician (Doctor of Medicine or Doctor of Osteopathic Medicine) or a dentist (Doctor of Dental Medicine or Doctor of Dental Surgery), (2) have a current US medical or dental license (ie, not revoked or expired), and (3) agree to complete and return the survey after reading the elements of informed consent in the cover letter.

### Recruitment

We purchased our sample from a licensee of the AMA and ADA who provided a systematic random sample of providers based on our selection criteria. The national sampling frame of practicing primary care and emergency medicine physicians provided by the licensee was obtained from the AMA Masterfile, the most commonly used and comprehensive sampling source for surveys of physicians in the United States [78-81]. Similarly, the sampling frame of dentists provided by the licensee came from the ADA, which maintains a list of active dentists in the United States. Our purchased sample frame from the licensee included provider name; type of degree; and any available contact information (postal address, telephone number, and email address).

We defined 6 different strata for our sample: AMA primary care physicians, AMA emergency medicine physicians, and dentists, with the members of each category identified by metropolitan and nonmetropolitan practice locations. Metropolitan and nonmetropolitan areas were defined using Metropolitan Statistical Areas, as defined by the Office of Management and Budget and used by the census. We specifically oversampled nonmetropolitan practice areas, given that there may be differences in provider stigma and attitudes, along with available treatment and referral resources, in nonmetropolitan regions compared with metropolitan ones [82] (see the section *Data Analysis Sample Size*). Systematic random samples were drawn from each stratum; every nth record was included in the sample based on the quantity requested versus the quantity available according to our specified criteria of provider characteristics. To assist our efforts to create sample weights for data analysis, the licensee provided the current population numbers for each stratum that we planned to sample.

To ensure that we only captured physicians and dentists currently in practice, we added a survey question on the cover page to assess whether the respondent was currently engaged in direct patient care. Although the AMA and ADA do track this information, we wanted to ensure that we omitted recent retirees or nonpractitioners. Within this survey question, we also asked whether the respondent planned to be in clinical practice for the next year. We chose to exclude those who planned to retire within the year, as their willingness to perform certain behaviors in the future (eg, receive future training programs and offer screening or treatment practices) may not be reflective of the current practicing workforce.

### Incentives

Providers were compensated for their time dedicated to completing the survey. In line with the Dillman [83] Total Design Method (TDM), each survey included an initial payment (US $10), regardless of survey participation, with the prenotification letter. Respondents who completed the survey then received additional compensation upon completion, which was gradually increased throughout the study. This methodology of a gradually increasing compensation was used in our prior national survey of dentists and helped substantially increase our response rate [73,84-86]. Furthermore, the amount of incentive that prompted a dentist to respond proved to be a significant covariate in that study’s primary outcome paper: dentists requiring enhanced incentives were significantly less likely to view expanded HIV screening (and other preventive health screenings) favorably as part of their professional role [86].

Participants were given options when selecting their method of compensation: an electronic gift card to 3 predetermined companies (Target, Walmart, and Amazon), a Visa debit card, or a mailed check. The participants also had the option of not receiving the compensation. The compensation scale upon completion of the survey (separate from the US $10 incentive in the first mailing) started with an initial payment of US $50. When the follow-up telephone prompting was initiated to recruit chronic nonresponders (ie, participants who had not responded to the initial recruitment attempts), we implemented an experimental design whereby the sample eligible for telephone prompting was randomized to receive an offer of either US $100 or US $150 upon completion (Table 1). After the US $100 and US $150 incentives were offered through the experimental design, we then offered chronic nonresponders an incentive of US $250 to complete the survey. This became necessary due to COVID-19–related challenges to the health care community, which caused staff shortages, large patient demand, and overburdened offices and hospitals; as such, increasing the incentive to US $250 helped compensate overly taxed providers who were experiencing significant clinical work burdens and time constraints during the pandemic. Notably, the availability of gradually increasing compensation time points (US $50, US $100, US $150, and US $250) will serve as important covariates in future study analyses.

**Table 1:** Design of the experiment.

Factor A: incentive offers in prompting	Factor B: prompter placing follow-up telephone calls	
	B1: NORC^a^ telephone prompter (control)	B2: study investigator (treatment)
A1: US $100 offer upon completion (control)	A1B1	A1B2
A2: US $150 offer upon completion (treatment)	A2B1	A2B2

^a^NORC: National Opinion Research Center.

### Study Procedures

Study procedures were implemented as a collaborative effort between the study investigators and the National Opinion Research Center (NORC). The NORC has administered leading social science surveys and has extensive experience in sampling design, survey data collection, recruitment of nonresponders, nonresponse bias analysis, and weight creation.

**Figure 1:**
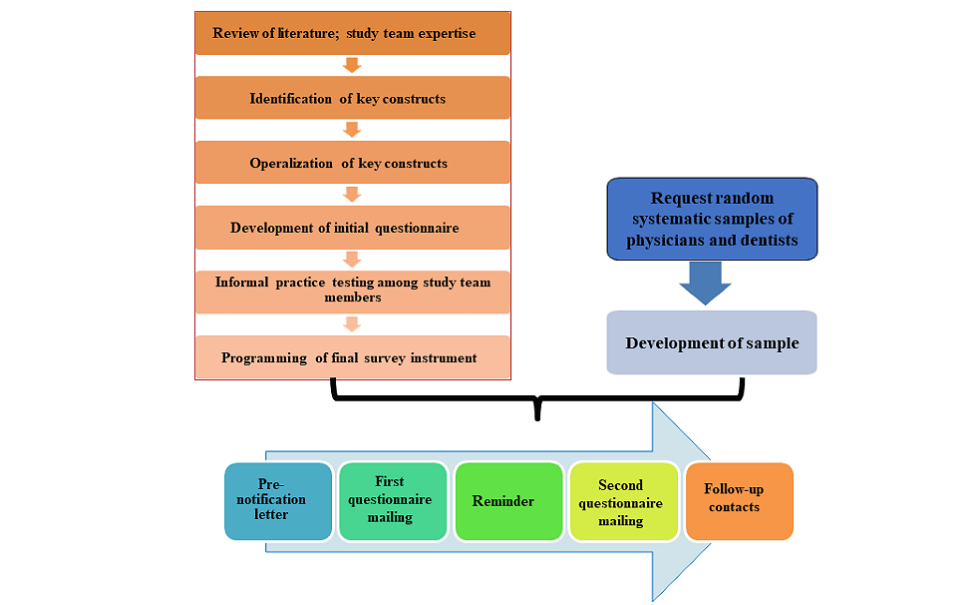
Study schema.

The overall methodology behind our study procedures was guided by the TDM, which implements strategies to help achieve a high response rate for survey studies (Figure 1) [83]. We followed the TDM-recommended series of contacts with participants in the following order: prenotification inquiry, initial questionnaire mailing (which included a link to a secure internet survey site), thank-you or reminder contact, replacement questionnaire, and follow-up telephone calls. Follow-up telephone calls to nonresponding providers were made with the goal of establishing personal contact, verifying the receipt of study materials, answering questions, and prompting for questionnaire return. In addition to the alternative options, respondents were given the option to complete the survey via telephone. These telephone calls were a collaborative effort between NORC staff (telephone interviewers) and the study investigators. Prior studies have shown some significant increases in response rates when physicians conduct follow-up prompting to other physicians [87-89]. Therefore, the telephone prompting phase was designed so that the sample eligible for telephone prompting was randomized to receive an initial follow-up prompting phone call from either an experienced NORC telephone interviewer or a study investigator (one with Doctor of Philosophy credentials and the other with Doctor of Dental Medicine and Doctor of Philosophy credentials). Similar to the case of our gradually increasing compensation, our future study analyses will have the option to include telephone prompting personnel (experienced NORC telephone interviewer vs study investigator) as a covariate (Table 1).

The initial study procedure protocols included offering the option of in-person completion, whereby NORC field managers would perform in-person visits to provider offices to prompt providers to complete and return the survey and offer providers the option to complete the survey in person with the field manager. However, due to the COVID-19 pandemic, we were not able to implement in-person study visits, given the restrictions implemented at hospitals and health care offices limiting visitors and guests, in addition to the travel-related transmission concerns that NORC staff would have encountered.

Participants were given multiple options to complete the survey. Internet- and mail-delivered surveys are strategies that have proven to be more cost-effective than traditional telephone surveys and are more compatible with health care providers’ constrained schedules [90]. Because the evidence comparing the effectiveness of mail- versus internet-based surveys is not conclusive, mixed-mode approaches that offer both methods of completion are generally common [91,92]. Mixed-mode approaches allow health care professionals to choose their preferred mode of response, which may be influenced by factors such as provider age, comfort with technology, personal contact, and security or privacy concerns [92,93]. As a result, the NORC continues to conduct many of their national survey studies using mixed-mode methods [94-99]. In addition, the task force of the American Association for Public Opinion Research recommends using a mail modality if using a single mode or a mixed-mode design that first uses mail contact and subsequently includes an internet-based response option [100].

We used other strategies endorsed by the TDM and supported by other studies to increase response rates, including prepaid incentives, personalized cover letters, and prepaid postage [101-103]. The survey’s self-completion methodology, use of a participant identification number, and specification (in the cover letter) that survey completion implies informed consent (in lieu of a signed consent) were additional methods used to minimize time constraints and maximize completion flexibility [104].

### Description of Study Assessments

#### Overview

The study assessment consisted of a 1-time cross-sectional survey instrument that, based on pilot-testing, required approximately 20 to 30 minutes to complete. Because research has shown that lengthier surveys result in lower response rates, especially among busy health care providers, brevity and reduced respondent burdens were both priorities in developing the survey instrument [101,105]. To ensure succinctness and respondent acceptability of our proposed survey instrument, we conducted informal practice testing among the key study team members and affiliates. The goal of this informal practice testing was to identify (1) the time required to complete the survey; (2) any questions that were confusing, difficult to interpret, inadequately written, or nonapplicable (logistical issues); and (3) any questions that were perceived as not important or too invasive (personal concerns). The feedback provided through this informal testing provided valuable input, suggestions, and edits that were reviewed by the study team for application to the final survey instrument.

The survey instrument administered to physicians differed slightly from that administered to dentists, reflecting the different scopes of practice for physicians versus dentists, along with differences in their expected training and backgrounds [106,107]. For example, survey questions querying about offering certain medical treatment practices (eg, prescribing MOUD and initiating antiretroviral therapy) were not included in the instrument disseminated to dentists. Notably, however, most measures were identical, including those assessing provider stigma.

The survey instrument included items related to providers’ attitudes and stigma. The Medical Condition Regard Scale (MCRS) [108] queried participants about their views on 3 categories of substance use (opioids, stimulants, and alcohol) as well as other medical conditions (type II diabetes, depressive disorder, and HIV) selected by the study team to assess the primary outcome. Specifically, these questions asked whether a provider views a given condition as enjoyable, treatable, and worthy of resources by asking them to report their agreement with a series of statements concerning a specified medical condition (eg, “Insurance plans should cover patients like this to the same degree that they cover patients with other conditions,” “Patients like this irritate me,” “I wouldn’t mind getting up on call nights to care for patients like this,” and “Patients like this are particularly difficult for me to work with”).

In addition to our primary assessment of stigma using the MCRS, we included additional measures of stigma in the attitude-assessing section, such as the Drug and Drug Problems Perception Questionnaire [109], which focuses on provider stigma in the form of provider “motivation” to treat patients with substance use disorders. We adapted other prior scales for appropriateness, such as the Link [110] study that assessed stigma in the form of general social distance and perceived dangerousness toward people with mental health disorders; in our survey, we instead assessed perceived dangerousness and social distance or isolation toward people with substance use disorders [110]. Other provider stigma measures related to persons with substance use disorders included general attitudes, attitudes toward providing medical or dental care, stereotypes, and familiarity. We also queried the providers’ personal history of substance use and treatment [109-120].

The instrument included many questions assessing providers’ clinical practices. To understand provider practices related to substance use treatment within the context of their overall practices, we asked participants how, if it all, they are addressing a myriad of different health care conditions that generally fall under the scope of primary care (and, in the case of dentists, general dental care): (1) type II diabetes, (2) depressive disorder, (3) HIV, (4) substance use, (5) alcohol use, and (6) tobacco or nicotine use. Of note, 5 of these conditions were queried in the MCRS section to assess providers’ attitudes toward these medical conditions. However, we collapsed opioid and stimulant use into 1 condition (“drug use”) and added tobacco or nicotine use to this section, as it is imperative to understand participants’ clinical practices related to forms of legal substance use (tobacco or nicotine), which can then be compared with their practices related to substance use. For each health condition, physicians were asked about their screening, treatment, and referral practices related to this condition; dentists were asked a similar set of questions but were not asked about providing treatment, given their differing scope of practice under their professional license.

Additional measures included social determinants of health (ie, whether providers are addressing patients’ social needs, such as homelessness, transportation, and interpersonal violence [121]); risk behaviors (in their personal lives and professional roles) [122]; subjective norms and social desirability (ie, their colleagues’ and patients’ perceptions of their professional role in substance use treatment); clinical knowledge (ie, prior education or training and future training needs in addressing substance use); clinical practice (ie, year of graduation, type of clinical setting, clinical discipline or specialty, patient insurance information, and patient population); and sociodemographics (ie, age, race, ethnicity, and gender).

#### Data Analysis Sample Size

The data analysis plan was developed to identify significant differences among the 3 subgroups of clinicians (primary care physicians, emergency medicine physicians, and dentists). In addition, we wanted to ensure that there was significant power to find clinically meaningful relationships in our planned secondary analyses. Primary care physicians were the largest planned subgroup within our study sample due to their diverse training backgrounds (n=1411), while emergency medicine physicians (n=800) and dentists (n=800) were also included as subsets of the population of health care providers in the general care workforce. The final target population was 3011, which was the target sample from which invitations to participate were sent. Allowing for a 70% response rate would provide an expected sample of 2108. The sample size needed to be further refined to account for the planned oversampling of nonmetropolitan clinicians. Oversampling resulted in slightly different design effects (Deff) within each of the other strata (primary care physicians: Deff=1.076, effective n=918; dentists: Deff=1.067, effective n=525; emergency department physicians: Deff=1.062, effective n=527). Applying the design effect induced by the oversampling of nonmetropolitan clinicians provides an expected effective sample of 1970 (primary care physicians: n=918, 46.6%; emergency medicine physicians: n=527, 26.8%; dentists: n=525, 26.6%). Power was calculated using PASS 2020 [123] with the expected effective sample sizes and a type II error rate of 20% (for 80% statistical power). We also report the power for a 50% response rate.

With our expected effective sample size, there is 80% power to construct CIs of the mean score on the MCRS with width of 0.088 SD of the MCRS. The CI width is 0.172 SD for dentists or emergency medicine physicians and 0.13 SD for primary care physicians. The intervals widen with a 50% response rate, ranging from 0.104 SD to 0.202 SD. When comparing MCRS scores between 2 different medical conditions, there is 80% power to uncover a difference when that difference is truly at least 0.06 of the SD for the full sample. This required true difference is 0.12 SD for dentists and emergency medicine physicians and 0.08 SD for primary care physicians. For a lower response rate of 50%, the required true differences to achieve 80% power move to 0.07 SD, 0.11 SD, and 0.14 SD for the full sample, primary care physicians, and emergency medicine physicians or dentists, respectively. When comparing MCRS scores between the different clinician types, there is 80% power to uncover a true difference that is 0.20 SD between dentists and emergency medicine physicians and when the true difference is 0.18 SD between either dentists or emergency medicine physicians and primary care physicians. The corresponding required effect sizes increase to 0.24 SD and 0.20 SD if the response rate is 50% rather than the targeted 70%. These effects are all considered small effect sizes.

Furthermore, the survey was designed to have sufficient power for secondary outcomes. To illustrate this, we used a binary secondary outcome (yes or no): “addressing substance use treatment (including providing substance use treatment/prescribing medications for OUD by physicians and screening and referral by dentists) = yes” versus “not addressing substance use treatment (not providing substance use treatment/not prescribing medications for OUD by physicians and screening and referral by dentists)= no.” Calculations were again made using PASS 2020 [123] with a 2-tailed test, α=.05, β=.2 (80% power). We assumed moderately conservative values of baseline probability (*P*_0_=.10 to .20) and *R*^2^ of 0.2 to 0.3 for other variables in the model and examined the impact of a 1 SD change in a continuous variable (such as the MCRS score) in predicting the binary outcome. With a 70% response rate, we have >80% power to uncover a significant effect if the true associated odds ratio is in the range of 1.19 to 1.29 for the full sample (1.23-1.35 if there is a 50% response rate). This range is 1.32 to 1.45 (1.36-1.55 for a 50% response rate) for primary care physicians and 1.41 to 1.63 (1.50-1.78 for a 50% response rate) for dentists or emergency medicine physicians. These effect sizes are in the small to moderate range.

#### Outcomes

Our primary outcome is providers’ “regard” for patients with substance use disorders, as measured by the MCRS [108], which will be assessed by 3 separate classes of substance use: (1) opioid use (including heroin, fentanyl, and nonmedical pain medication use); (2) stimulant use (including methamphetamine, cocaine, and crack use); and (3) alcohol use. These classes of substance use will be compared with providers’ “regard” toward the other assessed medical conditions. This MCRS measure is based on an 11-item scale that is scored as a continuous indicator, ranging from 11 to 66. Lower scores indicate “low regard” (ie, high stigma), while higher scores indicate “high regard” (ie, low stigma) for the given medical condition. This measure will be assessed for both physicians and dentists.

The secondary outcomes are specific to the provider type. For physicians, the secondary outcome will assess whether US primary care providers are currently offering on-site treatment for substance use (collapsed into 1 condition, “drug use”) in their clinical practice. For dentists, the secondary outcome will assess whether US dentists are referring patients for substance use treatment in their clinical practice. We are particularly interested in exploring the relationship between provider stigma toward persons with substance use disorders and practices related to addressing substance use in their clinical settings. The analyses of the outcomes related to physicians and dentists will be addressed separately.

#### Statistical Analysis

##### Overview

In general, statistical tests are determined by the distributional properties of dependent or outcome variables. The binary secondary outcomes will be tested using chi-square tests of independence for comparisons between groups and logistic regression for the multivariable case. For binary outcomes that are not rare, we will use Poisson regression with robust SEs to directly estimate the risk ratio (rather than the odds ratio, which diverges from the risk ratio when an outcome is not rare). According to the literature [62,108,124-126], we anticipate that “regard” will behave as a normally distributed continuous variable though the exact method of analysis will depend on the realized distribution of “regard” in the final survey data.

Our statistical approach will include descriptive statistics (mean, SD, and 95% CI) as well as mixed models and ANOVA to compare the regard for different medical conditions and across different practice settings, respectively. We will also model all outcomes as a function of a set of covariates and develop parsimonious models to identify provider characteristics associated with each outcome using the appropriate modeling procedure based on the outcome’s distribution (logistic regression for binary outcomes and linear regression for “regard”). All statistical analyses will use an α level of .05.

##### Item and Survey Nonresponse

Our first set of analyses will explore the potential biases that might arise if nonresponders differ significantly from responders along such dimensions as urbanicity. We may then explore the patterns of missing data. We would first derive binary measures that indicate whether the value of each variable for a respondent is missing. We would then relate these new measures to “key attributes” and consider statistically significant relationships as an indication of possible bias. Key attributes would be selected on the basis of (1) likely limited degrees of missing data and (2) substantive imports. If we find nonrandom patterns of item nonresponses, we will use multiple imputation methods [127] to minimize potential resulting biases. In addition, we will examine variables such as race, gender, and region in our nonresponse analysis to assess representativeness. If necessary, we would consider using nonresponse weighting to ensure that the results reflect the population.

### Data Management

Data collection activities were overseen by the NORC. All the invited providers were assigned a unique study ID number in their personalized cover letters; to ensure confidentiality, the cover letter emphasized the confidentiality of their survey participation, and their responses would not be linked to their identification. Any documentation linking provider information with the assigned unique ID was stored securely, with the access limited to specified NORC study personnel.

The NORC information technology team oversaw the data entry technology involved in electronic submissions, while production clerks were responsible for questionnaire receipt and library filing for those participants who completed the survey using paper (ie, self-administered paper questionnaire or fax. Once a completed self-administered paper questionnaire was returned to the NORC, it was sent to NORC’s Computer-Assisted Data Entry vendor for data entry and subsequent posting on a secure File Transfer Protocol site.

### Study Leadership and Investigator Procedures

This study was a collaborative effort among the lead Clinical Trials Network (CTN) study team, NORC, and National Institute on Drug Abuse (NIDA). The lead study team is a varied group of investigators with depth and breadth of experience in substance use disorders or treatment, internal medicine, infectious diseases, dentistry, epidemiology, public policy, social sciences, study training and supervision, provider surveys, and quality assurance monitoring. The NORC, founded in 1941, is one of the nation’s well-respected survey organizations. Their widespread staff members have extensive experience in achieving a high response rate in surveys, especially those of health care professionals, as well as in sampling design, recruitment of hard-to-reach participants, nonresponse bias analysis, and weight creation, which helped ensure that the best data were obtained for our national survey. Regular biweekly conference calls were conducted via videoconferencing between the study teams and the NIDA Scientific Officer throughout the study period to discuss survey activities, recruitment, and progress.

## Results

The study protocol was approved by the CTN Protocol Review Board in April 2020 and the UM IRB in August 2020. The survey instrument was officially mailed to the study sample in October 2020. Although we originally anticipated a 1-year follow-up period for data collection, our timeline was ultimately extended to 2 years due to unexpected disruptions caused by the COVID-19 pandemic. As such, data collection officially ended in October 2022, with an overall Council of American Survey Research Organizations rate of 53.62% (1240/2312.7; physicians overall: 855/1681.9, 50.83% [primary care physicians: 506/1081.3, 46.79%; emergency medicine physicians: 349/599.8, 58.2%]; dentists: 385/627.1, 61.4%). Note that the ineligibility rate among those screened is applied to those not screened, causing denominators to include fractional numbers. These rates resulted in a sample of 506 primary care physicians, 349 emergency medicine physicians, and 385 dentists.

Data analysis for the primary outcome paper, along with multiple planned secondary analyses, is currently underway, with plans to disseminate study findings in subsequent manuscripts in peer-reviewed journals. Findings from the analyses will be disseminated to CTN investigators, community partners, and representatives of the National Institutes of Health/NIDA. The planning, preparation, and submission of publications will follow the policies of the CTN Publications Committee. In addition, we plan to share our findings with a broad scientific audience through presentations at local and national conferences.

## Discussion

### Summary

Health care providers who practice primary care, emergency medicine, and dentistry are increasingly being called upon to help meet the growing demand for substance use treatment that surpass the availability of services [16,30]. Given their regular contact with patients with substance use disorders, these providers are well positioned to deliver and expand substance use treatment within their clinical settings [34]. However, before we can bridge this gap, we must first systematically and comprehensively understand the role of stigma in US health care providers’ treatment and clinical management of patients with substance use disorders.

Within this context, our survey findings assessed a wide range of attitudes, practices, and backgrounds of providers practicing throughout the nation. Our sampling methodology and high response rate have produced a particularly robust data set that will allow us to identify the factors, such as clinical setting and description of patient populations, related to provider stigma and the current state of substance use treatment delivery, as stated in our specific aims. In addition, our use of the MCRS will allow us to understand the magnitude and extent of provider stigma by the type of substance (opioids, stimulants, and alcohol). These 3 classes were specifically selected for not only being the most frequent types of substance use but also for being detrimental to individual and population health through overdoses, substance-related violence, and substance-related motor vehicle crashes [128-130]. Moreover, the availability of treatment modalities for these 3 classes differs; there are treatments for both OUD and alcohol use disorders [131], but recent research findings offer promise for stimulant use disorder treatment [132]. Assessing these 3 types of substance use will significantly contribute to the body of literature that assess provider stigma against substance use by substance class and how substance-specific stigma is associated with their respective screening and treatment clinical practices.

Furthermore, our study findings allow us to define provider stigma toward substance use in the context of other commonly stigmatized health conditions. The range of other conditions that we queried included different somatic, mental, and behavioral conditions (type II diabetes, depressive disorder, and HIV). Different chronic conditions can solicit different stigmas based on their visibility and perceived individual responsibility in causing the condition [133,134]; thus, it is important to select a range of chronic conditions that represent these differing manifestations and associations. In the case of HIV, provider stigma not only encompasses attributes such as prejudicial attitudes and discriminatory actions but also mortality-related judgment (due to values related to sexual practices and risk behaviors associated with HIV transmission) and fear of physical contagion from occupational exposure [135,136]. By contrast, provider stigma studies focusing on obesity and type II diabetes have shown provider stigma to be related to negative perceptions of patient behavioral factors (eg, laziness, lack of self-control, and dishonesty) [137,138]. In the context of mental health disorders, other constructs related to provider stigma include social distance (ie, self-reported willingness to engage and socialize with someone who has a mental health disorder) and perceived dangerousness (ie, the perception that people with mental health disorders are violent and should be feared) [25,139-141]. In the case of both HIV and mental health disorders, providers who elect to treat patients with these conditions have been shown to be particularly susceptible to “courtesy stigma” (ie, the subjective norms of other professionals), as colleagues and social relations question their willingness to treat patients perceived as socially or morally objectionable, which can impact providers’ social and professional reputation and also contribute to provider stigma [135,142-146]. Allowing for comparisons to be made between these chronic conditions, alone and in comparison with the substance use classes, is especially critical for comprehensively capturing the lens through which health care providers view and relate to the very patients they are being called upon to treat.

The limitations of our study include the cross-sectional nature of our methodology, which limits our ability to make causal inferences or assess temporal relationships. In addition, as the survey queried provider attitudes toward conditions that are commonly stigmatized as well as potentially sensitive topics (eg, prior substance use history of family, friends, colleagues, and self), there is potential for information bias in our study findings in the form of social desirability. However, our use of a participant ID and our security of a waiver eliminating the need for a signed informed consent form was specifically implemented for confidentiality purposes, which may have mitigated any reluctance to respond to the survey truthfully.

### Conclusions

Our survey was designed to identify barriers to addressing the overdose epidemic as faced by primary care physicians, emergency medicine physicians, and dentists who workforces called upon to change the way substance use treatments are delivered nationally and across patient populations. Our results can be used to inform future public health campaigns and provider-based interventions that provide new models of care, demonstrating the efficacy of initiating medication-assisted treatment in these settings [147,148]. By understanding providers’ subjective norms, the ways in which they view and address substance use, and their attitudes toward patients with substance use disorders, we plan to inform the development and implementation of stigma-reduction interventions that address providers’ perceptions and treatment of substance use. Although some effective interventions reduce stigma overall [149], the development of interventions specific to primary care and emergency medicine physicians and dentists requires a more granular understanding of substance-related stigma across the primary health care spectrum, particularly in settings that frequently encounter patients with substance use disorders.

## Abbreviations

ADA: American Dental Association
AMA: American Medical Association
CTN: Clinical Trials Network
Deff: different design effects
IRB: Institutional Review Board
MCRS: Medical Condition Regard Scale
MOUD: medications for opioid use disorder
NIDA: National Institute on Drug Abuse
NORC: National Opinion Research Center
OB/GYN: obstetrics/gynecology
OUD: opioid use disorder
TDM: Total Design Method
UM: University of Miami

## Data Availability

This study complies with the National Institute of Health Data Sharing Policy and Implementation Guidance [150]. Deidentified study data will be available to the public at the National Institute on Drug Abuse data repository, per the National Institute on Drug Abuse Clinical Trials Network policy. Details of data sharing can be found on the website [151].
